# *DMPK* hypermethylation in sperm cells of myotonic dystrophy type 1 patients

**DOI:** 10.1038/s41431-021-00999-3

**Published:** 2021-11-15

**Authors:** Shira Yanovsky-Dagan, Eliora Cohen, Pauline Megalli, Gheona Altarescu, Oshrat Schonberger, Talia Eldar-Geva, Silvina Epsztejn-Litman, Rachel Eiges

**Affiliations:** 1 Stem Cell Research Laboratory, Medical Genetics Institute, Shaare Zedek Medical Center, Jerusalem, Israel; 2 Hebrew University Medical School, Jerusalem, Israel; 3 Inserm UMR 974, Sorbonne Université, Association Institut de Myologie, Centre de Recherche en Myologie, Paris, France; 4 Zohar PGD Lab, Medical Genetics Institute, Shaare Zedek Medical Center, Jerusalem, Israel; 5 IVF Unit, Shaare Zedek Medical Center, Jerusalem, Israel

**Keywords:** DNA methylation, DNA methylation

## Abstract

Myotonic dystrophy type 1 (DM1) is an autosomal dominant muscular dystrophy that results from a CTG expansion (50–4000 copies) in the 3′ UTR of the *DMPK* gene. The disease is classified into four or five somewhat overlapping forms, which incompletely correlate with expansion size in somatic cells of patients. With rare exception, it is affected mothers who transmit the congenital (CDM1) and most severe form of the disease. Why CDM1 is hardly ever transmitted by fathers remains unknown. One model to explain the almost exclusive transmission of CDM1 by affected mothers suggests a selection against hypermethylated large expansions in the germline of male patients. By assessing DNA methylation upstream to the CTG expansion in motile sperm cells of four DM1 patients, together with availability of human embryonic stem cell (hESCs) lines with paternally inherited hypermethylated expansions, we exclude the possibility that *DMPK* hypermethylation leads to selection against viable sperm cells (as indicated by motility) in DM1 patients.

## Introduction

Myotonic dystrophy type 1 [DM1, (OMIM 160900)] is an autosomal dominant muscular dystrophy that results from a trinucleotide CTG repeat expansion (50–4000 copies) in the 3′ UTR of the *dystrophia myotonica* protein kinase gene (*DMPK*) [1, 2]. When the CTG tract expands beyond 300 repeats, they lead to abnormal CpG methylation next to the repeats in a way that is age and tissue dependent [3, 4]. The disease is classified into four [5] or five [6] somewhat overlapping forms, which incompletely correlate with expansion size in somatic cells of patients. With rare exception, it is affected mothers who transmit the congenital (CDM1 >1000 CTGs) and most severe form of the disease [7–9].

Why CDM1 is hardly ever transmitted by fathers remains unresolved. One potential explanation would be frequent oligo/azoospermia among male patients with the adult form of the disease leading to reduced fecundity [10]. This may be in addition to behavioral changes that often result in male patients less frequently acquiring partners in particular populations [11]. However, both factors are not sufficient to explain the acute tendency toward maternal CDM1 transmissions. A different explanation for the underrepresentation of paternal CDM1 cases may be the absence of very large expansions in the sperm of DM1 males. Indeed, while exceedingly small mutations are prone to further expand in the male germline, larger mutations (>80 repeats) often contract in a way that negatively correlates with expansion size [12]. Hence, although the role of male transmission is to trigger repeat instability at low mutation range (as exemplified by excess of transmitting grandfathers among children with the congenital form of the disease [13]), for larger mutations it most likely limits allele length by yet to be identified factors/mechanism(s).

Previously, Barbe et al. reported rigorous data concerning a strong correlation between aberrant methylation immediately upstream to the CTG repeats [CTCF1 site], and maternal transmission of CDM1 by the analysis of a large cohort of DM1 patients [14]. In light of their findings, a model that relies on the systematic elimination of particularly large mutations by hypermethylation upstream to the repeats in the male germline was proposed to explain the unresolved question of why CDM1 is almost always transmitted by affected mothers [14]. This model is especially attractive since it parallels the observed involvement of a similar mechanism in Fragile X syndrome [4, 15–17].

**Fig. 1:**
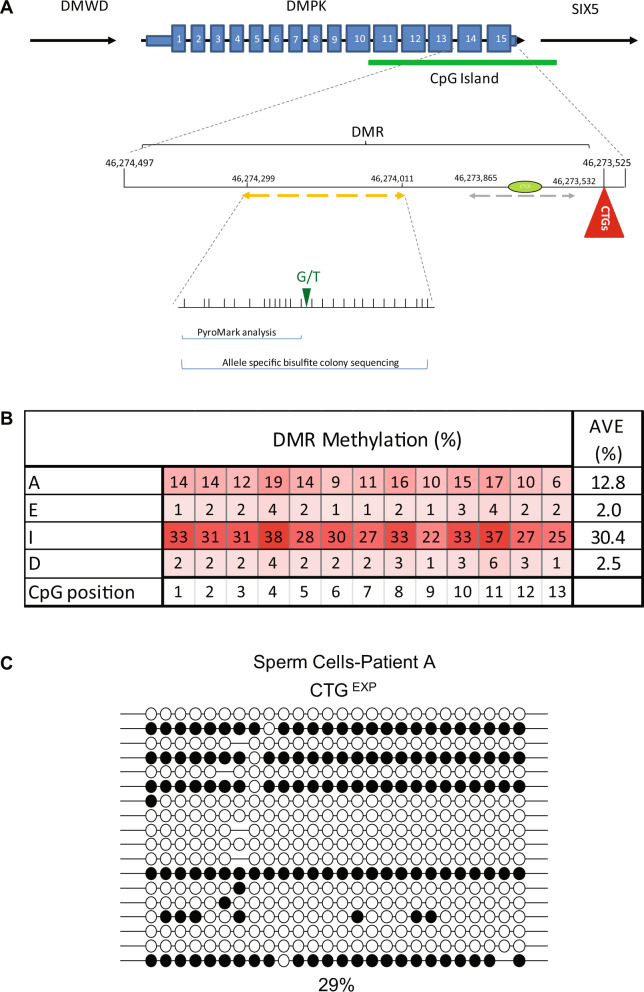
DM1 sperm cell methylation analysis. **A** Graphic illustration of the human DM1 locus, including *DMWD*, *DMPK*, and *SIX5* genes. Indicated in detail the differentially methylated region (DMR) upstream to the CTG repeats. Dashed line in orange designates the amplicon that was used for the methylation analysis in this study covering SNP rs635299 (G/T), while dashed line in gray designates the amplicon that was used for the methylation analysis in Barbe et al. [14]. CTCF1 binding site is designated by green ellipse. The numbering represents chromosome 19 positioning according to the hg19 human genome assembly. **B** Absolute methylation levels in region E (488–777 bp upstream of the CTGs), as determined by bisulfite pyrosequencing. The 13 CpG sites tested are indicated by shaded squares according to methylation levels (see colored legend). Mean absolute methylation levels across all tested CpG positions are indicated to the right in each line. **C** Allele-specific bisulfite sequencing of the expanded allele (CTG^EXP^) within the differentially methylated region of *DMPK* (region E in [4]), overlapping SNP2 (according to [4]), in DNA extracted from sperm sample of patient A. Full circles correspond to methylated CpGs, whereas empty circles represent unmethylated CpGs. Methylation levels (%) were determined by the analysis of 20 molecules by bisulfite colony sequencing. Methylation levels (29%) are indicated at the bottom.

To test this hypothesis and determine the timing of *DMPK* hypermethylation in DM1, we tested sperm from four male patients undergoing preimplantation genetic diagnosis (PGD) procedures for DM1 (Table 1, IRB 110/11 followed by IRB 90/17) (for methods see Supplementary Material). Sperm isolation was carried out by a two-layer gradient system which is designed to guarantee that the sample is predominately composed of motile sperm with normal morphology. Methylation analysis was carried out on residual sperm samples by DNA bisulfite massive parallel sequencing (pyrosequencing). In this set of experiments, CTG repeat allele size could not be determined simultaneously with methylation status because residual sperm cells were available to us only in small quantities. Nevertheless, we assayed methylation levels in a restricted region located 650 bp upstream to the CTG repeats (13 CpG sites) (see Fig. 1A) which was formerly identified as a disease associated DMR (Differentially Methylated Region) that becomes hypermethylated in a way that best correlates with the inherited size of the mutation (progenitor allele length) and putatively acts as a regulatory element for *SIX5* transcription [4]. In addition, because methylation systematically and uninterruptedly spreads from the upstream flanking region toward the repeats, methylation status 650 bp upstream to the CTGs (referred to as region E in ref. [4]) could be directly inferred from, and thus compared to, alleles that are heavily methylated at the CTCF1 binding site (the region analyzed by Barbe et al.). Hence, we aimed our methylation analysis at region E and found abnormal methylation levels of 13% and 30% (corresponding to 26% and 60% on the background of the expanded allele) in viable sperm cells of patients A and I, respectively (see Table 1 and Fig. 1B). Taking advantage of an informative SNP which resides within the DMR sequence (rs635299), we further validated the results in patient A by bisulfite allele-specific colony DNA sequencing, demonstrating methylation levels of nearly 30% exclusive to the mutant allele (Fig. 1C, altogether 26 CpG sites, bisulfite conversion rate nearly 100%). Of note, methylation levels among these patients did not correlate with sperm quality (Table 1). In addition, it should be mentioned that all four patients are now fathers to at least two healthy children following PGD procedures.

**Table 1:** Detailed description of sperm donations.

DM1 patient	Age	CTG copies^a^	Sperm total motile count (TMC)	Sperm quality (volume, sperm count/ml, motility, morphology)	Fertilization rate by ICSI	Average levels of methylation in sperm	DM1 hESC lines (CTG copies)^b^
A	35	N/A	2 × 10^3^–5 × 10^2^	Severe OTA (3 ml, <1 × 10^6^, 10%, 2%)	63–67%	13%	SZ-DM1 (1000) SZ-DM2 (430, 500, 1000) SZ-DM6 (1060) SZ-DM11 (500) SZ-DM12 (630) SZ-DM20 (300)
E	37	700	18.8 × 10^6^	OTA^d^ (5 ml, 3 × 10^6^, 30%, 2%)	92–100%	2%	SZ-DM7 (300)
I	28	1000	16 × 10^6^	Teratoospermia (2 ml, 80 × 10^6^, 66%, 1%)	61%	30%	–
D	31	~300^c^	200 × 10^6^	Normospermia (5 ml, 24 × 10^6^, 50%, 10%)	100%	2.5%	SZ-DM5 (300)

*N/A* data not available.

^a^CTG copy number was determined in blood samples of all patients and human embryonic stem cell (hESC) lines by fragment length analysis of restriction digested genomic DNA resolved by agarose gel electrophoresis and detected by Southern blot hybridization with a DIG-labeled probe, excluding patient A, who was diagnosed by linkage analysis. The estimated allele lengths (determined by the modal allele length from the dense part of the smear) are confounded by the age-dependent and expansion-biased nature of somatic mosaicism and thus may not reflect the inherited allele length.

^b^DM1 affected embryos were obtained in all patients during PGD procedures.

^c^Repeat length of patient D was determined by PCR analysis as described in ref. [20].

^d^Oligoasthenoteratozoospermia.

Although we analyzed a different region from Barbe et al. a region located a bit further upstream to the CTG repeats, our findings are consistent with upstream methylation not affecting spermatogonial stem cell death and/or the production of nonviable sperm cells, thus narrowing the timing of selection, if at all, to not before fertilization. On the contrary, it is apparent that paternal hypermethylated mutations are compatible with embryo viability, at least until the embryo implantation stage. This stems from the availability of a considerable number of hESC lines which we established from affected embryos with a paternally inherited mutation (some derived from patient A in Table 1) that are methylated along the entire region upstream to the repeats, including the region that is designated as a CTCF binding site (referred to CTCF1 in ref. [14] or region F in ref. [4]). Importantly, in DM1 hESCs, methylation levels and spreading are totally dependent on expansion size and not parental origin of the mutation, provided that the number of the repeats is >300 [4]. The larger the mutation, the farther methylation spreads from intron 13 of *DMPK* toward the CTGs. Yet, considering that all of the hESC lines were established from intracytoplasmic sperm injection (ICSI)-derived embryos (as is regularly carried out in all PGD cycles), we may have bypassed a natural barrier while producing these embryos by assisted reproduction techniques (ART) i.e.,; the failure of mutant hypermethylated mature sperm cells to fertilize an oocyte or to trigger embryonic genome activation after penetrating the egg [18].

Therefore, all things considered, the data presented here suggest that the stem/mature sperm cell selection model is one of the following: (i) incorrect, (ii) leaky, and (iii) correct except that haploid sperm containing methylated expansions are fertilization incompetent or are unable to trigger embryonic genome activation because of methylation, expansion size or some other yet identified paternal factor. Although the dynamics of hypermethylation in the early embryo may be a bit different so that hypermethylation is first erased and then reestablished (as opposed to directly inherited from the sperm), this should not conflict with the claim that the suggested model for sperm cell selection by upstream methylation is most likely incorrect. Alternative models for maternal transmission of CDM1 by sperm cell selection should be sought such as, but not limited to, differences in the activity level of mismatch proteins [19] or other repeat length controlling modifiers among male and female germlines.

For further investigation, we suggest to extend this study, which was limited to a very small number of individuals, to a much larger cohort of patients. In addition, progenitor mutations in *DMPK* should preferably be classified by size, age and purity of the repeats to better substantiate our findings. Moreover, it would be informative to follow the dynamics of hypermethylation along with expansion size during other stages of spermatogenesis. Furthermore, assessment of the competence of sperm cells with methylated alleles to fertilize an egg without the involvement of ART is warranted in order to gain more insight into the timing and mechanisms underlying these biological events.

## Supplementary information

Supplementary material

## Data Availability

The datasets generated and analyzed during the current study are available from the corresponding author on reasonable request.
