# Supplementary material for: DMPK hypermethylation in sperm cells of myotonic dystrophy type 1 patients
Source: Eur J Hum Genet. 2021 Nov 15;30(8):980–3. doi: 10.1038/s41431-021-00999-3 (PMC9349176; doi:10.1038/s41431-021-00999-3)
Supplement: Supplementary file 1 — Supplementary material [file 41431_2021_999_MOESM1_ESM.docx]

Methods

Sperm Isolation

Sperm preparation from single ejaculates was carried out using ISolate® Sperm Separation Medium (FUJIFILM Irvine Scientific, cat #99264), which is based on a density gradient medium designed to separate the motile fraction of semen from the seminal fluid. Next, sperm cells were collected into a separate drop for ICSI according to their normal motility and morphology via aspiration into an ICSI needle. Following the ICSI procedure, the remaining sperm cells in the drop were collected for the study.

SNP Analysis

Genomic DNA from each subject was amplified using hemi-nested PCR (forward primer: CCTGTCCCTTCTCTCTCCAG and reverse primers: CATTCCCGGCTACAAGGAC and GTCATTGGCTGCTTCCTAGC), and compared with parental DNAs at the SNP site (rs635299) to determine whether informative and to determine allelic association with the CTG expansion (as described in Yanovsky-Dagan et al. (4)).

Bisulfite Sequencing

Genomic DNA (2 μg) was modified by bisulfite treatment (EZ DNA methylation kit, Zymo Research) and amplified by FastStart DNA polymerase (Roche). Amplified products (289 bp) were cloned, and single colonies were analyzed for CpG methylation by direct sequencing (ABI 3130). For pyrosequencing, PCR products were analyzed using PyroMark Q24 (QIAGEN). The primer set for the bisulfite pyro-sequencing was: TGGTTGTGGGTTAGTGTT and Biotin‐CCCAACAACCTACAACTATTAT.

Analysis of Expansion Size by PCR

To estimate the number of CTG repeat length in patient D by PCR, DNA from blood was amplified by PCR according to (22) with small modifications. In brief, 5 ng of genomic DNA was amplified in a 25 ul reaction using primers ST300F (5’-GAACTGTCTTCGACTCCGGG-3’) and ST300R (5’- GCACTTTGCGAACCAACGAT-3’) using 1X Custom master mix (Thermo Fished Scientific, Courtaboeuf, France) and 0.06 units of Thermoperfect *Taq* Polymerase (Peak International products b.v., LZ Earbeek, Netherlands) under the following conditions: denaturation at 96^0^C for 5 minutes followed by 30 cycles of 96^0^C for 45 seconds, 60^0^C for 30 seconds and 72^0^C for 3 minutes, followed by one cycle of 60^0^C for 1 minute and a final extension step at 72^0^C for 10 minutes. Amplified products were analysed by gel electrophoresis on agarose gel of 0.8%. The approximate number of repeats was calculated by subtracting 361 bp from the length of the PCR product divided by 3.
